# Mathematics Competence Level: The Contribution of Non-symbolic and Spatial Magnitude Comparison Skills

**DOI:** 10.3389/fpsyg.2019.00465

**Published:** 2019-03-05

**Authors:** Marisol Cueli, Débora Areces, Ursina McCaskey, David Álvarez-García, Paloma González-Castro

**Affiliations:** ^1^Department of Psychology, University of Oviedo, Oviedo, Spain; ^2^Center for MR-Research, University Children’s Hospital Zurich, Zurich, Switzerland; ^3^Children’s Research Center, University Children’s Hospital Zurich, Zurich, Switzerland

**Keywords:** comparison skills, mathematics competence, non-symbolic comparison, preschool children, spatial comparison

## Abstract

Magnitude comparison skills have been related to mathematics competence, although results in this area vary. The current study aimed to describe the performance of 75 children (aged 4–5 years) in two comparison tasks; and examine the strength of the relationship between each of the two tasks and mathematics competence level (MCL). Participants were assessed with the Early Numeracy Test which provides a global MCL score. Magnitude comparison skills were assessed with two tasks: a non-symbolic number comparison task and a spatial comparison task. Results of the Pearson correlation analysis showed a relationship between the two tasks with better performance in the spatial comparison task. Regression analysis with the stepwise method showed that only the non-symbolic number comparison task had a significant value in the prediction of the MCL pointing to the need to take these kinds of tasks into account in the first years of school.

## Introduction

A prominent characteristic of the majority of modern societies is the ubiquitous role of numeracy in conducting day-to-day activities (e.g., shopping or traveling requires the ability to make decisions based on quantitative information; Gilmore et al., 2013). Mathematical skills are therefore crucial abilities in modern life (Ancker and Kaufman, 2007) and early individual differences in mathematics have been reported to predict later adult socioeconomic status (Ritchie and Bates, 2013). Given this prominence, it is important to increase our knowledge of the cognitive processes underlying children’s achievement in mathematics.

Findings from the Primary International Assessment Exercises which assess academic performance (International Association for the Evaluation of Educational Achievement [IEA], 2011; Organisation for Economic Co-operation and Development [OECD], 2014) warn about the existence of mathematical learning difficulties in children. Despite adequate and age-appropriate achievement in other educational domains, approximately 6–14% of school-age children have persistent difficulties with mathematics (Barbaresi et al., 2005; Clayton and Gilmore, 2015).

The study of cognitive determinants related to mathematical skills can be analyzed from either a domain general or a domain-specific perspective (Fias et al., 2013; Bellon et al., 2016). Domain general approaches focus on non-numerical cognitive skills that play a role in mathematical performance, including executive functions such as working memory, processing speed, and inhibition control (Friso-van den Bos et al., 2013; Gilmore et al., 2013). Domain-specific approaches study the role of number-specific processes, such as individual differences in the representation of numerical magnitudes (Schneider et al., 2016). Domain-specific skills considered to be central to mathematics include procedural competence, conceptual understanding, counting, number fact knowledge, and Approximate Number System (ANS) acuity or “number sense” (Baroody, 2003).

### The Approximate Number System (ANS)

The ANS is a pre-linguistic cognitive system for representing and processing quantity information and has received a great deal of attention in recent years (Dehaene, 1997; Cantlon et al., 2009; Gallistel, 2011; Gilmore et al., 2014; Leibovich et al., 2017; Peng et al., 2017; Cai et al., 2018; Odic and Starr, 2018). The ANS supports the representation and processing of different magnitudes (Cantlon et al., 2009) and according to Dehaene (1997), it is a universal system present in animals, children, and adults.

Studies have shown that adults and children are able to use this system to compare and order sets of items presented as arrays of dots (Feigenson et al., 2004; Barth et al., 2005). The ANS allows comparison, addition, and subtraction of quantities without counting them (Dehaene, 1997). According to Odic et al. (2016), the ANS has three main characteristics: (1) Discrimination performance in the ANS is ratio-dependent based on Weber’s law (discriminating a collection of 12 items from six items is easier than discriminating a collection of 12 items from 11 items); (2) there are large individual differences in ANS precision (the ANS improves from birth until around age 30); and (3) the ANS has been located in both the human brain and in non-human animals (specifically, the ANS is associated with a region of the intraparietal sulcus).

An individual’s ANS acuity can be measured empirically with various tasks. These include symbolic (e.g., digit) or non-symbolic (e.g., dot) approximate comparison and addition tasks, or estimation tasks which assess the mapping between symbols and non-symbolic representations.

The most commonly used measure of ANS acuity is a dot comparison task, involving the comparison of two non-symbolic visual arrays of dots (Odic and Starr, 2018). During this task, participants see two dot arrays and must estimate which array they believe has more dots in it and respond either by key press, verbally, or by pointing. The response format used generally depends on the presentation methods employed and the age of the participants, and the performance is often indexed by accuracy (i.e., how often the participant correctly selects the more numerous array; Clayton and Gilmore, 2015). Performance on dot comparison tasks is affected by a distance effect and a size effect (Holloway and Ansari, 2010). The distance effect refers to the observation that decisions are more difficult when the numerical distance between the stimuli is small (in relation with the ratio-dependent effect). The size effect reflects more difficult discriminations for numerically larger numbers. This influence of task characteristics in how children make decisions about number has been known since Piaget’s research, in which children erroneously judged one line of objects as more numerous when the objects were spaced further apart (Piaget, 1952). Starr et al. (2017) found that numerical decision-making in 4–6 year olds and adults was influenced by non-numerical features and when participants in their study were attempting to make decisions based on the numerosity of the arrays, even adults were unable to ignore the spacing of items within the arrays (although this effect decreased significantly with age).

The precision of the ANS in making non-symbolic comparisons improves with age (Halberda et al., 2008; Gómez-Velázquez et al., 2015; Odic et al., 2016), and has been proposed as a precursor of mathematical skills. Also, according to Odic and Starr (2018), the ANS is more precise in some people than in others and these individual differences emerge early in development and stay relatively stable with age (precision at 6 months predicts precision in preschool). Furthermore, individual differences in ANS precision demonstrate a small but significant relationship with formal math, including in preschoolers (Feigenson et al., 2013; Odic et al., 2016) and also correlate with the level of mathematics achievement (Halberda et al., 2008). For example, meta-analyses have reported significant correlations between ANS and mathematics (Chen and Li, 2014; Schneider et al., 2018) and many studies have shown a predictive association between number comparison skills and mathematical achievement (e.g., Piazza et al., 2010; Feigenson et al., 2013; Sasanguie et al., 2013; Starr et al., 2017). Starr et al. (2017) found that numerical acuity (measured with a non-symbolic comparison task) was the strongest predictor of variance in math achievement (although many other factors such as IQ and executive functions must be taken into account). Mazzocco et al. (2011) found that poor performance in non-symbolic approximation tasks distinguishes children with mathematical learning disabilities from their typically performing peers. Bonny and Lourenco (2012) found that preschoolers (3–5 years of age) with more precise number representations were generally more mathematically competent, as assessed by a standardized test of early math achievement. However, results in this area vary (Feigenson et al., 2013; Kroesbergen and Leseman, 2013) and not all studies have found significant links between non-symbolic number performance and mathematics achievement in children (e.g., Vanbinst et al., 2012; Kroesbergen and Leseman, 2013; Sasanguie et al., 2013). These differences in research findings could be related to the kind of tasks used to assess comparison skills.

One important issue that could affect performance in non-symbolic tasks is related to the dimension or representation of magnitude. Many researchers have suggested that number, time, and space are all represented by common mechanisms “a domain-general generalized magnitude system” (Odic et al., 2016). Walsh (2003) proposed a theory of magnitude (ATOM), which asserts that time, space, and number are all processed by this common magnitude system, located in parietal brain regions. Additional evidence for the generalized magnitude system comes from correlations of Weber fractions across dimensions and from persistent congruency and interference effects between quantities, whereby manipulation of one dimension affects discrimination performance of another (Odic et al., 2016). However, authors such as Henik et al. (2017) suggested that the ability to perceive and evaluate sizes or amounts might constitute a more primitive system that has been used throughout human evolution as the basis for the development of the number sense and numerical abilities. How children and adults discriminate between different magnitudes has been analyzed from two perspectives: (1) the relationship between non-symbolic comparison, spatial comparison, and mathematics achievement and (2) the relationship between the performance in tasks with different magnitude systems (i.e., non-symbolic comparison and spatial comparison).

From the first perspective, Cai et al. (2018) examined the effects of symbolic (number line task) and non-symbolic estimation (point comparison task) on mathematics skills across three grade levels (kindergarten, children from grade 2, and children from grade 4). Their results showed that in kindergarten, non-symbolic estimation predicted all early mathematics skills while in grades 2 and 4, symbolic estimation accounted for unique variance in mathematical problem solving, but not in calculation fluency. Authors suggested that different types of ANS acuity should be used to predict mathematics skills in different learning periods and perhaps to identify children at risk of having difficulties in mathematics. Lourenco et al. (2012) tested the extent to which estimations of numerical and non-numerical magnitudes predicted math competence in college students. The tasks consisted of deciding which of two dot arrays was larger in either numerical value or cumulative surface area. Participants’ accuracy scores on both magnitude tasks were positively correlated with performance on tests of advanced arithmetic. Later, Lourenco and Bonny (2017) used this procedure with 67 students between 5 and 6 years old who completed two magnitude comparison tasks (judge which of two discrete arrays was larger in numerical value and judge which of two amorphous displays was greater in cumulative area). They found that performance on number and area comparison tasks correlated with performance on exactly the same math tests and representations of cumulative area, and predicted children’s math performance.

From the second perspective, Kucian et al. (2018) looked at the association between discrete non-symbolic number processing (comparison of dot arrays) and continuous spatial processing (comparison of angle sizes) in 367 children between the third and sixth grade. Their findings suggested that the processing of comparisons of dots and angles are related to each other, but angle processing was easier in their sample, so they concluded the existence of a more complex underlying magnitude system consisting of dissociated but closely interacting representations for continuous and discrete magnitudes. For this work, they used a task described in a previous study (McCaskey et al., 2017) which included a non-symbolic and a spatial task. Both tasks required a magnitude judgment, which is either based on discrete quantity estimation of numerosity (number) or on continuous spatial processing (space). However, other authors such as Agrillo et al. (2013) did not find correlations between non-symbolic estimations (number/space/time) in 35 adults between 19 and 32 years old, which contradicts the existence of a general magnitude system.

Given the interest in the results of previous research (Agrillo et al., 2013; Cai et al., 2018; Kucian et al., 2018), and that Cai et al. (2018) found different performance in kindergarten and primary school, it would be useful to analyze the task set in McCaskey et al. (2017) in children younger than 6 years old and compare the results with those from Kucian et al. (2018) in older students between 8.2 and 12.9 years of age.

### The Present Study

The intention of the present study is to look deeply at the performance of preschool children when they have to make a magnitude judgment. Izard and Spelke (2009) showed that sensitivity detecting relationships of line length and angles improves over childhood, until 12 years of age. Furthermore, Starr et al. (2017) found that while 4-year-old children’s numerical judgments were most influenced by non-numerical features, 6-year-old children exhibited strikingly adult-like performance, which suggested to these authors that numerical decision-making undergoes substantial change between 4 and 6 years of age.

With this in mind, this study aims to: (1) describe the performance of preschool children (aged 4–5 years) in the two magnitude comparison tasks used by McCaskey et al. (2017) and Kucian et al. (2018) and (2) examine the strength of the relationship between each of the two tasks (non-symbolic and spatial magnitude comparison) and mathematics competence level (MCL).

## Materials and Methods

### Participants

Participants in this study were 75 students enrolled in three second-year kindergarten classes, in the Principality of Asturias (North of Spain). Schools were public and were located in a city-center. By law, classes must have no more than 25 students per class. All the families reported a medium-high socio-economic level and consisted of three to four members.

The students were aged between 4 and 5 years old (*M* = 52.47, *SD* = 3.91 months; in a range of 46–59 months). Of these students, 44 (59%) were girls and 31 (41%) were boys. There were no statistical differences in the gender-distribution of boys and girls in the current sample, *χ^2^*(1) = 2.25, *p* = 0.13. Furthermore, differences in the MCL were not significant in terms of age (*p* = 0.228), gender (*p* = 0.836), or intelligence quotient (IQ; *p* = 0.275) according to univariate analysis of variance. A convenience sample was recruited for the study. Written informed consent was obtained from the parents of the participants of this study. No children had been diagnosed with learning disabilities and all of them had an IQ between 80 and 130 (IQ *M* = 99.52; *SD* = 14.99).

### Measures

#### Raven’s Colored Progressive Matrices

Raven’s progressive matrices provide a non-verbal assessment of intelligence. The test offers three progressively more difficult forms intended for different populations. Items on all forms ask the examinee to identify the missing component in a series of figurative patterns. In this study, the colored form (CPM; Raven et al., 1996) was used. This is used to assess children from 4 years of age. It consists of 36 items in three sets of 12. The administration time is usually 15–30 min.

#### Early Numeracy Test Revised

The original revision of the Early Numeracy Test – Revised (ENT-R; Navarro et al., 2009; Mendizábal et al., 2017) was completed by Van Luit and Van de Rijt (2009) and subsequently standardized for the Spanish population (Van Luit et al., 2015). The ENT-R evaluates early numerical knowledge and aims to detect students with mathematical learning disabilities. This tool is especially useful in the transition from preschool to elementary education. It can be used to confirm which students need support to cope with the new mathematical learning, thereby promoting the implementation of early intervention procedures. The test assesses eight skills: concepts of comparison, classification, one to one correspondence, seriation, verbal counting, structured counting, counting (without pointing), general knowledge of numbers, and estimation. A global MCL score can be obtained based on performance across the eight subtests. The ENT-R is suitable for children aged 4–7 years. There are three parallel versions of 45 items each, version A was used in the current study. It takes an average of 30 min to complete the test, which is individually administrated. Previous studies have reported a Cronbach’s alpha reliability index of 0.95 (Mendizábal et al., 2017). In the current study, the Cronbach’s alpha was 0.76. Only the global MCL score was used for analyzing the relationship with the two magnitude comparison tasks.

#### Magnitude Comparison Tasks

Comparison skills were assessed by the test developed by McCaskey et al. (2017). It is based on two tasks: a non-symbolic number comparison task and a spatial comparison task. Both tasks require a magnitude judgment, which is either based on the evaluation of discrete quantity estimation of numerosity (number) or on continuous spatial processing (space). The first task is based on the presentation of two sets of dots. Children have to indicate on which side more black dots are presented. The second task shows a green and a blue pacman facing each other with varying mouth sizes. Children have to indicate which of the two pacman figures has a bigger mouth. The first is a non-symbolic number comparison task, and the second requires a visuo-spatial and continuous magnitude decision.

**FIGURE 1:**
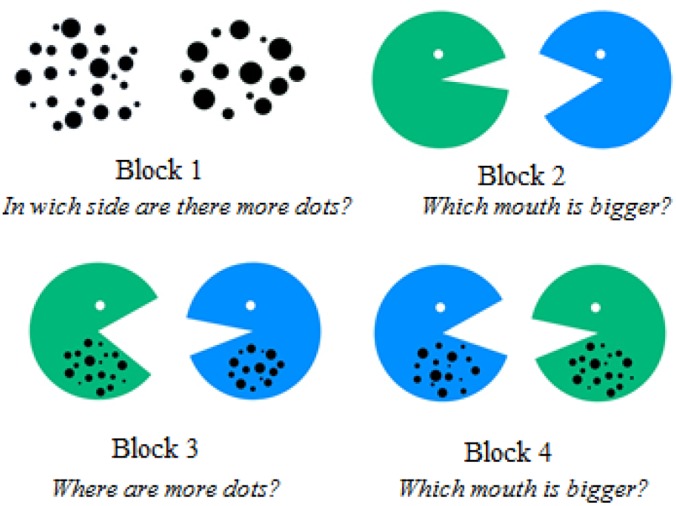
Example of one trial for each block presented to the students. They have to answer 20 questions for each block.

The tasks were presented using E-prime software (Version 2.0). There were 80 different trials, classified into four blocks (20 trial per block). In the first block “dots” (B1), in each trial two groups of dots ranging from a minimum of 12 to a maximum of 30 dots, were presented horizontally. Children were asked to indicate on which side more black dots were presented (Figure 1). Presentation of dots was controlled for individual size of dots (no judgment possible due to individual dot size), total displayed area (no judgment possible due to total black area), distribution of dots (no judgment possible due to total covered area), and the numerical distance between presented magnitudes. All children were carefully introduced to the task and encouraged to solve all trials by comparison of both sets of presented dots by numerical estimation and highlighting the importance of not counting. Responding was indicated by pressing a key corresponding to the side of the larger magnitude (z key or m key).

In the second block “mouths” (B2), a green and a blue pacman facing each other with varying mouth sizes were presented horizontally. Children had to indicate which of the two pacman figures had a bigger mouth (Figure 1). In contrast to the non-symbolic number (dot) comparison task, this task required a visuo-spatial and continuous magnitude decision. The mouth angle of the pacman figures varied between a minimum of 27° to a maximum of 68°. The side of the correct answer and color of correct pacman were balanced. In the same way as for the number comparison task, children were carefully instructed and advised to solve the spatial comparison task by simple estimation of mouth sizes and not to use other approaches (e.g., their fingers, or any other tool) to measure the mouth sizes.

For the third and fourth blocks, the stimuli were combined and each presentation consisted of a green and a blue pacman facing each other with the dots presented inside the figures (Figure 1). In the third block “dots combined” (B3), the child was required to decide in which of the two sets there were more dots. In the fourth block, the child had to indicate which mouth size was bigger. In the third block, nine trials were congruent (more dots and a bigger mouth) and 11 trials were incongruent (fewer dots and a bigger mouth). Similarly, in the fourth block “mouths combined” (B4), there were nine congruent (a bigger mouth and more dots) and 11 incongruent trials (bigger mouth and fewer dots). The same stimuli were used for blocks 3 and 4, although order of presentation was randomized. Children were explicitly instructed to look at the dots (block 3) or at the mouths (block 4).

All tasks were administered in an untimed format following the procedures from other authors such as Defever et al. (2013) and Szücs et al. (2013). An untimed task allowed us to complete all the trials and avoid omissions in performance. Finally, the ratio between smaller and larger dot arrays and between smaller and larger mouth angle across all blocks was 0.5, 0.6, 0.7, 0.8, and 0.9. The same ratios were used in the four blocks but the order of presentation was randomized. In summary, the first and third blocks were based on a non-symbolic number comparison task and the second and fourth blocks were spatial comparison tasks. Prior to the start of each block, students performed four training trials with ratios of 0.5 and 0.6 to ensure that they understood the instructions. All students did the same training trials and received feedback during this initial practice.

### Procedure

After obtaining research approval (the study was approved by Ministry of Science, Innovation and Universities of Spain and by the University of Oviedo, Asturias, Spain), local preschools were randomly selected and approached to take part in the study. The schools forwarded the information about the study to parents of the children with a request for informed consent. The IQ of the children whose parents agreed to participate was assessed with the Raven’s CPM. All children scored an IQ between 80 and 130 and were therefore included in the study, undergoing further testing with the ENT-R and the comparison task. All the assessment tasks were administered by qualified educational psychologists and were coordinated and guided by the same educational psychologist from the research group.

The study was conducted in accordance with the Declaration of Helsinki (World Medical Association, 2013). The evaluations were carried out over consecutive days during regular classes.

### Data Analysis

Preliminary examination of the data showed that the assumptions (e.g., skewness and kurtosis) required for the use of parametric statistics were met. All analyses were conducted using SPSS for Windows Version 22. Differences were considered significant at level of *p* < 0.05. For both the non-symbolic number and the spatial comparison tasks, the accuracy or correct responses (CRs) were taken into account (CRs over total items).

Initially, to describe the performance on the two comparison tasks, Pearson correlation coefficients were calculated. In order to study this relationship in depth, the CR in every block was compared by paired student *t*-tests and effect sizes were calculated. For the interpretation of the effect sizes, Cohen (1988) criterion was used, which establishes that the effect is small when ηp2 = 0.01 (*d* = 0.20), medium when ηp2 = 0.059 (*d* = 0.50), and high if ηp2 = 0.138 (*d* = 0.80).

Second, to examine the strength of the relationship between each of the two tasks and MCL, a hierarchical multiple regression analysis was carried out. We tested three models. The MCL was included in the analysis as the dependent variable. In the first model, gender, age, and IQ were used as independent variables; in the second model, the CR in block 1 (B1) and block 3 (B3) were added as independent variables (given that these blocks are based on a non-symbolic number comparison task); and in the third model, the CR in block 2 (B2) and block 4 (B4) were taken also included (given that these blocks are based on a spatial comparison task).

## Results

### Pearson Correlation Coefficients

The correlations are provided in Table 1, including the mean, standard deviation, skewness, and kurtosis of the four blocks (dots, mouth size, dots combined, and mouths combined).

**Table 1:** Correlation matrix of the magnitude comparison skills and MCL including means, standard deviations, skewness, and kurtosis.

	1. Block1	2. Block2	3. Block3	4. Block4	5. MCL
1. Block 1	–	0.24^∗^	0.34^∗∗^	0.09	0.40^∗∗∗^
2. Block 2		–	0.31^∗∗^	0.37^∗∗^	0.21
3. Block 3			–	0.28^∗^	0.27^∗∗^
4. Block 4				–	–0.16
5. MCL					–
*M*	13.07	17.20	13.11	17.47	22.05
*SD*	2.65	1.74	2.63	2.22	14.87
Skewness	0.05	–0.54	0.04	–1.97	1.24
Kurtosis	–0.63	0.02	–0.44	5.78	2.19
Minimum	8	13	7	8	1
Maximum	19	20	19	20	76


*^∗^p < 0.05, ^∗∗^*p* < 0.01, ^∗∗∗^*p* < 0.001. *Note.M* = mean; *SD* = standard deviation; efficacy = correct responses – incorrect responses; MCL = mathematics competence level provided by ENT-R; Block1 = comparing dots; block 2 = comparing mouth sizes; block 3 = comparing dots showed inside pacman mouths; block 4 = comparing pacman mouth sizes with dots inside.*

As can be seen in Table 1, significant correlations were found between B1–B2, B1–B3 and between B2–B3, B2–B4, B3–B4. Also B1 and B3 showed a significant relationship with the MCL. In Table 2, the percentages of CRs are provided.

**Table 2:** Proportion of correct responses for the four blocks of the comparison task across the five ratio conditions.

Comparison task	Ratio				
	0.5	0.6	0.7	0.8	0.9
Block 1	68%	44%	21.%	15%	5%
Block 2	93%	73%	72%	64%	13%
Block 3	77%	65%	51%	51%	3%
Block 4	81%	89%	69%	66%	53%


*Note. Block 1 = comparing dots, block 2 = comparing mouths; block 3 = comparing dots combined; block 4 = comparing mouths combined.*

The *t-*test showed significant differences between B1–B2 *t*(74) = -12.76, *p* < 0.001, *d* = 2.1; B1–B4 *t*(74) = -11.50, *p* < 0.001, *d* = 1.89; B2–B3 *t*(74) = 13.29, *p* < 0.001, *d* = 2.18; B3–B4 *t*(74) = -12.85, *p* < 0.001, *d* = 2.11. Differences were not significant between B1–B3 (*p* = 0.910) and B2–B4 (*p* = 0.312). Student performance was similar in the comparison of dots (B1) and dots inside the mouth (B3) and also in the performance in the comparison of mouths (B2) and mouths with dots inside (B4) which makes sense given the common nature of the tasks. The means indicate more CR in the execution of B2 and B4 showing more CRs when children have to compare mouths than when they have to compare dots. Furthermore, independently of the presentation of dots alone or inside the pacman mouth, the performance was similar with better results in B3 (dots inside the pacman) which could be related to the congruence effect. Similarly, the performance for the presentation of the pacman mouth alone or with dots inside did not differ.

### Regression Analysis

In a second step, we carried out hierarchical multiple regression analyses in order to analyze which of the comparison skills better predicts the MCL.

The MCL was taken as dependent variable and the CR of the four blocks as independent variables. In a first model, gender and age were used as independent variables. In the second model, age, gender, and CR of blocks 1 and 3 were included as independent variables. These two blocks assess non-symbolic comparison skills. Finally, in the third model, the CR of blocks 2 and 4 were introduced in addition to gender, age, CR of blocks 1 and 3. These two blocks assess spatial comparison skills. Results showed that the three models were significant with *F*(3,71) = 3.299, *p* = 0.025; *F*(5,69) = 3.928, *p* = 0.003, and *F*(7,67) = 4.060, *p* = 0.001, respectively. In the first model, IQ was significant (*p* = 0.032). In the second model, B1 was significant (*p* = 0.019) and in the third model B1 (*p* = 0.024) and B4 (*p* = 0.010) were significant.

Looking at *R*^2^ and the adjusted *R*^2^ for the three models, 12% of the variation in MCL can be explained by model 1, 22% by model 2, and 29% of the variation in the MCL can be explained by model 3 (see Table 3).

**Table 3:** Hierarchical regression analysis models.

	Model 1		Model 2		Model 3	
	β (β standardized)	*t*	β (β standardized)	*t*	β (β standardized)	*t*
Age	0.73(0.19)	1.71	0.48(0.12)	1.16	0.36(0.09)	0.89
Gender	–3.36(–0.11)	–1.00	–2.01(–0.06)	–0.61	–1.65(–0.05)	–0.52
IQ	0.26(0.24)	2.18^∗^	0.16(0.15)	1.37	0.16(0.15)	1.40
B1			1.58(0.28)^∗^	2.40^∗^	1.47(0.26)^∗^	2.31^∗^
B3			0.65(0.11)	1.00	0.94(0.16)	1.43
B2					1.24(0.14)	1.22
B4					–1200(–0.30)^∗^	–2.66^∗^

aR2	0.122		0.222		0.298	
ΔR2	0.085		0.165		0.224	


*^∗^*p* < 0.05. *Note*. Values in the table are the non-standardized β regression coefficient; those in brackets are the standardized values. *t* = student *t*-test; R2 = variance explained; ΔR2 = change in variance explained; B1 = correct responses of block 1 comparing dots; B2 = correct responses of block 2 comparing mouth; B3 = correct responses of block 3 comparing dots combined; B4 = correct responses of block 4 comparing mouths combined.*

## Discussion

The aim of this study was to analyze the performance of 4 year old children in two magnitude comparison tasks, a non-symbolic and a spatial comparison task. Furthermore, we were interested in examining which of the two tasks was more strongly related with MCL.

Results showed that both tasks were significantly, positively related when dots or mouths were shown alone (B1 and B2) or combined (B3 and B4). The correlation between the blocks was 0.24 and 0.28. Kucian et al. (2018) obtained a very similar result with a correlation of 0.26 between the two tasks and 0.25 controlling for the effect of age and grade level. These authors were also interested in whether the strength of this correlation decreased with development. They observed that in fifth grade, the correlation was weaker than in lower grade levels, but they did not observe differences in sixth grade regarding previous levels. If we compare the 4-year-old students (in this study) with the third to sixth grade students (in Kucian et al., 2018) the correlations are very similar and *a priori*, they would not yield significant differences. In short, if we consider present and past results we can see that both tasks are significantly related to each other.

However, at the same time, we found differences in the performance in every task. Regarding the mean performance of the students, it seems that the non-symbolic comparison task was more difficult than the spatial comparison task. Students made more mistakes when comparing dots alone or inside the mouths. This result is in line with research from McCaskey et al. (2017) who showed a significant relationship between the two tasks and pointed out that when the ratios were similar (as in the present study), spatial judgment of angle size is easier compared to non-symbolic magnitude comparison. Leibovich and Henik (2013) pointed to higher accuracy levels for a continuous spatial task compared to non-symbolic dot comparison. Odic et al. (2013) showed higher acuity for continuous spatial processing (comparison of area sizes) than non-symbolic number processing (comparison of dot arrays) in 3–6-year-old children. Also, Kucian et al. (2018) found that in third to sixth grade students, spatial comparison was generally easier than non-symbolic number comparison. They showed significant differences between the number and spatial tasks in third, fourth, fifth, and sixth grades.

These differences in both tasks are also reflected in the performance in the four blocks with respect to the ratios, which were the same in the four blocks (0.5, 0.6, 0.7, 0.8, and 0.9). In this case, we saw performance decrease as the ratio approached 1 and in consequence, the level of difficulty increased. The decrease is more evident in the case of the non-symbolic comparison task than in the spatial comparison task. Odic et al. (2016) highlighted that discrimination performance in the ANS is ratio-dependent based on Weber’s law (the accuracy of this system varies as the quantity increases, with the comparison being easier for very different sets such as 10 versus 5). These results are also in line with previous work by Kucian et al. (2018), in which the accuracy levels decreased significantly for both conditions (non-symbolic magnitude comparison task and spatial comparison task) with increasing ratio between magnitudes (bigger ratios mean smaller distances between magnitudes and are therefore more difficult to compare). The authors hypothesized, similarly to Leibovich and Henik (2013), that the superiority of processing continuous magnitudes might indicate that this system is older than the system for processing discrete magnitudes and might develop earlier during childhood than the discrete quantity system. Our results point in the same direction and support this idea of previous and older development of the continuous magnitude system, although more research is still needed. Our second aim was to examine the strength of the relationship between each of the two tasks (non-symbolic and spatial magnitude comparison) and the level of mathematics competence (MCL). The correlation analysis showed that there was a relationship between the non-symbolic comparison and the MCL showing that the child’s performance in this type of task is related to their level in mathematics but not performance in the spatial task in this sample. This has an immediate educational implication. When teachers analyze the performance of their students in comparison activities, it is very important for them to take into account performance in non-symbolic comparison tasks because that could be more related to their mathematics level in this age range. In this sense, the results in the dots comparison task are compatible with the findings of Mazzocco et al. (2011) or Feigenson et al. (2013) who highlighted that poor performance on non-symbolic approximation tasks distinguishes children with mathematical learning disabilities from their typically performing peers. The results from the spatial comparison task, which did not show a correlation with the MCL, differ from previous research (Lourenco et al., 2012; Cai et al., 2018). This could be related to the difficulty level given that although the two tasks in the study were comparable in their design (same ratios for the non-symbolic and spatial comparison task), that did not mean the same level of difficulty for 4-year-old children, and the second task was easier for them so it is possible that it did not discriminate sufficiently. This could be the reason for the absence of a relationship with the MCL reflected in the regression analysis.

The regression analyses showed that the dots comparison alone had a significant value in the prediction of the MCL (model 2). The mouths combined comparison was also significant in the explanation of the MCL (model 3). However, the dots comparison seems to have more weight in this prediction given that the value in B1 (dots comparison) was positive while the value in B4 (mouths combined comparison) was negative. In any case, this supports the results found by Feigenson et al. (2013) and Sasanguie et al. (2013) showing a predictive association between number comparison and mathematical achievement. The mouths combined comparison exhibited a relationship with MCL, albeit negative. This result could be associated with the characteristics of the task. The second and fourth blocks included congruent and incongruent trials. The total number of trials in each block was 20, and this may not have been sufficient for accurate assessment when the two situations are included (nine congruent and 11 incongruent). The negative result is quite surprising, and needs to be replicated in the future, in order to understand whether the reason is associated with the congruent and incongruent trials, or whether children could be looking at other characteristics of the stimuli, or even whether this task is especially difficult for 4-year-old students. Children can answer by looking at the dots instead of the angles in the fourth block and for this reason, they may answer incorrectly in the incongruent trials (when the bigger angle has fewer dots). Starr et al. (2017) highlighted that performance is typically better for congruent trials compared to incongruent trials and the effect of congruency is strongest for young children and attenuates with age, suggesting that younger children may be more biased by non-numerical cues than older children. The influence of the congruence and incongruence effects could be the reason for the children’s better performance in block 3 compared to block 1. In this sense, it is possible that tasks including congruence and incongruence for students between 4 and 5 years old are not appropriate to their level and do not provide significant information. However, this needs to be studied more in the future.

Taking the results together, we can see that comparison at 4 years old can be influenced by different aspects (magnitude used, congruency, characteristics of the stimuli as the density or size) that make it harder to interpret the children’s performance. Non-symbolic comparison tasks (such as the dots comparison) may be more useful with simple designs including ratios lower than 0.7, given that ratios of 0.9 are extremely complicated for children at this educational level. However, in spatial comparison tasks (such as the mouths comparison), lower ratios are especially easy for children and the design of the tasks have to include ratios higher than 0.7 to improve discriminatory power. In addition, the use of congruency and incongruency has to be studied more deeply and could be analyzed in relation with the MCL. It is important to note that in this study we used congruency and incongruency in terms of their relationship between the mouths and the dots (more dots and a bigger mouth or fewer dots and a bigger mouth). Typically, congruency has been studied in terms of the features of the stimuli and considering trials congruent trials when one or more visual cues (dots area, density) are positively correlated with numerosity, and incongruent trials, when one or more visual cues are negatively correlated with numerosity. Several studies have demonstrated that these visual cues can influence numerosity judgments such as Gebuis et al. (2009) and it has been associated with other factors such as inhibition (Szücs et al., 2013), so it could be interesting to analyze the profile of performance in the task in relation to the executive function levels.

Finally, this study has the following limitations that must be taken into account. First, sample selection by accessibility is a limitation of the study, although it is necessary given the difficulty of going into the schools and working with children as it affects the running of the school. Also, it is necessary to note that the sample size is rather small for multiple regression analysis but it allows us to draw preliminary conclusions in this line of research using this specific comparison task. However, given that the aim of the study was to determine the strength of the relationship between the MCL and the numerical and spatial tasks, and given the differences between the two tasks and the MCL, it would be useful to check these results in students of these ages and even to use more trials for each block of tasks to avoid possible ceiling effects. In addition, the MCL was taken as a global measure rather than using specific mathematic skills (classification, seriation, one to one correspondence, verbal counting,…), it could be interesting in the future to examine the relationship of each specific mathematical skill to the two comparison tasks. In any case, in conclusion, the results of our study have a practical implication for teachers, showing that tasks associated with the comparison of dots could provide an approximate measure of students’ MCL. At the same time, activities that require that comparison can enhance and improve students’ MCL, so it might be interesting to incorporate these kinds of tasks in the objectives and instructional procedures for teaching mathematics in preschool. In short, even from the first years, teachers can have an approximation of a student’s MCL and improve it directly or indirectly through tasks of magnitude judgment such as the comparison of dots.

## Author Contributions

MC, DA, and PG-C contributed to the design of this study. UM contributed to the design of the comparison task. MC and PG-C organized the data collection and database. DÁ-G performed the statistical analyses. MC and DA wrote the first draft of the manuscript. UM and PG-C wrote a major revision of the manuscript. All authors contributed to manuscript revision, read, and approved the submitted version.

## Conflict of Interest Statement

The authors declare that the research was conducted in the absence of any commercial or financial relationships that could be construed as a potential conflict of interest.
